# Teaching medical trainees to see societal infrastructure as a clinical issue

**DOI:** 10.3389/fmed.2025.1686996

**Published:** 2025-11-11

**Authors:** Waseem Jerjes, Marcin Klingbajl, Azeem Majeed

**Affiliations:** Department of Primary Care and Public Health, Faculty of Medicine, Imperial College London, London, United Kingdom

**Keywords:** infrastructure, medical education, health systems science, interprofessional teamwork, clinical advocacy

## Infrastructure determines health: the clinical evidence

Medical training traditionally focuses on detecting pathology through history-taking, physical examination, imaging, and laboratory testing. However, clinical outcomes are often shaped as much by the built environment as by biomedical factors. Problems such as inadequate housing, poor sanitation, unreliable transport, and unstable energy supply are all linked to increased morbidity and mortality. In England, more than 11% of households experience fuel poverty, contributing to excess winter deaths and exacerbating chronic illness (1, 2).

Globally, the World Health Organization reports that 24% of all deaths are directly caused by modifiable environmental and infrastructural determinants (3). Taken together, these data establish infrastructure as a clinical determinant rather than background context. The question, then, is why curricula rarely make infrastructure a routine part of clinical reasoning.

## The hidden curriculum of infrastructure

Despite this evidence, undergraduate and postgraduate teaching rarely embeds infrastructure within history-taking or clinical reasoning (4, 5) In the UK medical curricula, infrastructural deficiencies are frequently mentioned as “social determinants of health” but rarely as part of clinical reasoning nor taught as key targets for appropriate intervention (6). This gap aligns with Health Systems Science (HSS) in US medical education, which frames learners as “systems citizens” and includes social determinants, systems thinking, and advocacy as core competencies (7, 8). For example, common issues such as cold, damp housing and unreliable transport regularly shape presentation and adherence, particularly for disadvantaged and rural populations (9).

Clinical education thus unintentionally embeds a hidden curriculum, implicitly teaching students to disregard infrastructure as outside clinical responsibility. Repeated omission cements bifurcated patient care with clinicians competent at short-term clinical management but less aware regarding systemic infrastructural determinants of recurrent illness (10–12).

Comparable curricular gaps are reported internationally—from US settings (e.g., food access) to low- and middle-income contexts where power reliability affects life-saving care (13–16). The consequences of this are profound. Students learn a restricted concept of their clinical role, limiting their ability to act progressively on their patients' behalf or to recognize when clinical interventions will fail on their own (17). Newly qualified practitioners graduate ill-equipped to handle or to recognize upstream determinants of ill health, making them less effective and maintaining health inequities (18–20).

Medical educators must accept this latent curriculum and look instead to openly incorporating infrastructural issues into clinical education. Trainees must learn early on to respond to and recognize infrastructural failure as part of clinical practice proper and not as ancillary issues of public health. Only thereby will future doctors truly reach the determinants of the health of their own patients (21–23).

## Infrastructure as a clinical emergency

Transport infrastructure failures hinder healthcare access, causing significant numbers of missed medical appointments annually in England, particularly in rural and disadvantaged areas (24). There is measurable clinical impact: delay in cancer diagnosis, poorer control of chronic disease, and increased emergency hospital attendances (25, 26). Rural Scotland, for instance, has had higher cardiovascular and cancer mortality rates as a direct result of delay and interrupted therapy due to inadequate transport connectivity (27).

These are repeated globally and enhance the global scope of infrastructural determinants of health. In sub-Saharan Africa and globally, inadequate sanitation contributes to over 564,000 diarrhoeal deaths annually, most of which are preventable with improved infrastructure (28). In rural India, frequent electricity outages severely disrupt essential healthcare services such as dialysis and neonatal intensive care, substantially compromising patient safety and clinical outcomes (29). In American cities, minority groups disproportionately face environmental hazards, such as contaminated water supplies, with high-traffic examples including Flint, Michigan, wherein infrastructural neglect directly caused clinical instances of toxicity to lead (30–32).

Clinically, these deficits present as missed appointments, delayed diagnoses, medication failures and preventable exacerbations—patterns that perpetuate avoidable harm (24, 27, 29, 33, 34). Recognizing these patterns as clinical emergencies clarifies thresholds for action beyond symptomatic care when infrastructure is the proximal driver of harm.

## Integrating infrastructure into medical curricula

To truly respond to health inequities related to infrastructure, curricula for health professionals will need to transform to teach students to see infrastructure not as a contextual factor but as an essential clinical predictor of the health of the patient (1, 7, 35).

Having established infrastructure as a clinical determinant, we now turn to what must change in formal training. In clinical training today, there is minimal focus on the external environment and the direct impact it and the supporting infrastructure have on disease propagation and outcomes for the patient. Medical students are constantly taught to carefully review specific organ systems individually, but they are often not taught to systematically review environmental parameters like the quality of housing, access to transport, or energy security—elements as important to the wellbeing of the patient as are laboratory tests or radiology reports. The interventions below operationalize this shift within existing teaching blocks with minimal disruption.

This approach converges with the H&P 360 framework within Health Systems Science, which extends the history and examination to seven domains that include context, resources, and systems navigation. Incorporating our infrastructure prompts within an H&P 360–style template makes housing, energy security, transport, and water safety routine elements of clinical data-gathering rather than *ad-hoc* social notes (8).

The inclusion of an infrastructure approach as part of the curriculum of medicine requires practical modifications to clinical training (6, 36). An important one is to teach students to take comprehensive infrastructure-led histories as a routine. Just as they are instructed to routinely question the history of smoking habits, the history of the family's health, or drug regimen, they should just as routinely question the character of the patient's surroundings, household heating, access to safe drinking water, and consistency of power supply. Inclusion of such questioning early on normalizes the recognition of the significance of infrastructure as clinically relevant so that students will discover and thereby intervene against the environmental determinants of disease.

Another effective approach is using case-based learning scenarios to illustrate clearly how infrastructural deficits directly lead to clinical presentations (12, 20, 37) Examples such as an elderly person whose diabetes control is frustrated through limited access to healthy foods can vividly outline how infrastructural environments directly dictate clinical courses. Through the integration of such cases into the curriculum, educators can illustrate directly the correlation of infrastructural realities to disease control with clinical futility of repeated therapy in the absence of intervention on environmental determinants.

A successful method for framing the clinical significance of infrastructural deficiencies is the use of structured case-based learning exercises (1, 5, 9, 22, 38). These educational approaches map directly to H&P 360 domains—for example, infrastructure-focused history ↔ context/resources; community placements ↔ systems navigation; and advocacy skills ↔ teamwork and health systems improvement (8). Table 1 presents thorough examples illustrating direct correlations between ubiquitous clinical presentations and discrete infrastructural deficiencies to further establish the imperative of including conceptualization of the role of infrastructures explicitly as part of medical school curricula.

**Table 1:** Clinical presentations linked to infrastructure deficits (UK and global perspectives).

**Clinical presentation**	**Clinical implications**	**Typical clinical management**	**Infrastructure deficit (UK examples)**	**Infrastructure deficit (Global examples)**	**Potential solutions (UK and global)**
Frequent asthma exacerbations	Chronic lung damage, recurrent hospitalization, poor quality of life	Inhalers, corticosteroids, emergency admissions	Damp, poorly insulated housing affecting ~4 million UK homes	Poor-quality housing and indoor pollution (e.g., biomass fuel cooking) in India, Africa	Housing retrofitting, ventilation improvements, clean energy cooking solutions
Missed dialysis or chemotherapy appointments	Disease progression, increased morbidity and mortality risk	Rescheduling treatments, hospital admissions	Unreliable public transport impacting rural and disadvantaged UK communities	Limited rural transport—including many U.S. counties as well as sub-Saharan Africa and rural Asia—leads to missed appointments and poorer treatment adherence.	Enhanced public transport, subsidized patient transportation schemes
Poorly controlled diabetes	Cardiovascular and renal complications, hospital admissions	Medication adjustments, frequent hospital visits, dietitian referral	Food deserts in UK urban centers affecting deprived neighborhoods	Food deserts in U.S. cities and limited nutritional access in urban slums in India, Brazil, and South Africa.	Community food initiatives, subsidized healthy food access, urban agriculture programs
Medication spoilage (insulin, biologics)	Increased emergency admissions, poor disease management	Replacement prescriptions, acute hospital interventions	Sporadic electricity outages affecting vulnerable areas in the UK	Power instability—U.S. grid failures alongside frequent outages in sub-Saharan Africa and rural India	Stable power supply, backup generators, renewable energy investments
Increased diarrhoeal diseases and infections	Severe dehydration, malnutrition, high child mortality rates	Oral rehydration therapy, antibiotics, hospitalization	Localized sanitation issues in disadvantaged urban areas in the UK	Unsafe drinking water and poor sanitation infrastructure in sub-Saharan Africa, rural Asia	Improved sanitation, safe drinking water initiatives, infrastructure investment in clean water access
Mental health deterioration (stress, anxiety, depression)	Increased morbidity, decreased productivity, reduced quality of life	Antidepressants, counseling services, psychiatric care	Overcrowded, poorly maintained housing in urban UK areas	Severe overcrowding, lack of green spaces in rapidly urbanizing regions globally	Urban planning initiatives, improved housing standards, community green spaces
Lead poisoning and chemical exposure	Cognitive impairment, developmental delays, neurological damage	Chelation therapy, specialist referral	Historical cases of environmental contamination in UK industrial towns	U.S. Flint water crisis, industrial contamination in rapidly developing countries	Regular environmental monitoring, stricter regulatory frameworks, infrastructure remediation

Clinical rotations in communities directly affected by infrastructural deprivation similarly embody transformative teaching potential (13, 18, 30, 39). Medical schools can intentionally assign students to rural counties which lack public transport, socio-economically disadvantaged neighborhoods with shelter inefficiencies, or neighborhoods with healthcare infrastructural lacks. Such experiences not only demonstrate the determinant function of infrastructure on health outcomes but also oblige trainees to experience the profound frustration and powerlessness of the patient ensnared in patterns of preventable disease. Through immersion with the community directly affected, students can begin to learn empathy and moral accountability for infrastructural advocacy as part of their professional selves.

Educational modules that are truly interdisciplinary with practitioners from civil or urban planning and public health further enrich medical training (2, 9, 14, 40). Interprofessional learning allows students to see the value of alternate perspectives and innovative solutions beyond the standard model of medicine. For instance, architecture or urban planning workshops may expose trainees to innovative models of housing that were specially designed to promote respiratory health or to reduce the risk of falls in frail elderly populations. In these truly interdisciplinary exercises, students not only learn more but gain the confidence to engage productively in systemic advocacy with clear vision for the intersectionality between the built environment and clinical outcomes.

Ultimately, for such curricular innovations to bear lasting impact, medical schools ought to incorporate into clinical education itself a culture of advocacy (19, 30, 31, 34). Education for students on advocacy skills—how to present the evidence associating infrastructural gaps with clinical outcomes, how to frame the product of policy decisions, and how to collaborate with community stakeholders—instructs students with practical skills for executing real transformation. Cultivating reflective practice on advocacy interactions—both achievements and barriers overcome—can solidify the confidence and competency of trainees to confront infrastructural determinants across the span of their careers.

## Repositioning clinical responsibility

Curricular change must be matched by professional practice. Reframing clinical responsibility to include infrastructure requires broadening common understandings of medical professionalism (3, 8, 16, 29). Traditionally, clinicians understand their own work through narrow lenses, diagnosing and curing disease for individuals. Though critical, this perspective misses the larger structural determinants of the health of their patients. Clinicians regularly witness patients experiencing recurrent health crises stemming from external environmental conditions and yet often perceive these issues as not their responsibility. Breaking this narrow perspective is essential since clinicians are the only practitioners with the potential to bridge individualized patient care with social dimensions. Framed through Health Systems Science, this is the move from competent clinicians to effective “systems citizens” who can act on infrastructure when it is the proximal driver of harm (7).

One critical barrier is the demarcation between clinical practice and advocacy for public health (7, 17, 19, 26, 34). Clinicians already routinely walk social advocacy paths: arranging accommodation support for high-risk patients, advocating for benefits support, or assistance with disability advocacy. Elevating this existing advocacy to the level of infrastructure is then second nature. Clinicians are regularly directly exposed to the health implications of infrastructural abandonment. Viewing such infrastructural failings as tangible clinical issues, not distant policy matters, helps clinicians to look beyond fixation on symptoms to removal of the causative sources.

Repositioning clinical responsibility requires training for advocacy and systems-based practice (4, 7, 18, 27, 39). Medical education should systematically instruct students and trainees to investigate, record, and report health outcomes influenced by infrastructure. Structured advocacy module-based educational interventions, community-based practical attachments, or inter-disciplinary projects can considerably enhance the competency of trainees to engage effectively in structural advocacy. Training clinicians to present clinical evidence to decision-makers— highlighting, for instance, the direct relationship between damp housing conditions and chronic respiratory illnesses—enhances their power to influence health-related infrastructural choices.

Clinicians are highly qualified professionals with high social status, so they are especially well-suited to champion infrastructural investments (11, 17, 26, 32). Society values clinicians' knowledge highly and listens to them with respect when they talk about determinants of health. This respected position allows clinicians to initiate effective dialogue with policymakers, local government leaders, and community organizations and to directly correlate infrastructural conditions with measurable clinical outcomes. For example, clinician advocacy about air quality evoked policies to reduce rates of pollution in cites and then hospitalization for many respiratory diseases subsequently declined.

Ultimately, repositioning clinical responsibility to include infrastructure advocacy enhances rather than detracts from the clinician's professional role (2, 8, 17, 24, 40). It creates an ethical and practical responsibility for involving the holistic determinants of the health outcomes of patients. Through the restructuring of medical professionalism to include systemic advocacy, clinicians break cycles of preventable disease, reduce health inequities, and promote the attainment of genuine, long-lasting patient wellbeing. Such restructuring not only broadens the scope of clinical practice but also anchors medical care to its core moral imperative: to do no harm by addressing the determinants of disease.

To minimize role creep and protect wellbeing, responsibility is team-based rather than physician-centric. Physicians retain accountability for recognizing when infrastructure is the proximal driver of harm and for initiating an appropriate route, while the interprofessional team (nursing, physiotherapy, pharmacy, social worker, link workers) undertakes most actions, with public health, housing, planning and engineering engaged when hazards are recurrent or structural. This preserves feasibility, embeds advocacy within existing pathways, and develops “systems citizens” capabilities across professions—not only doctors.

## Sharing responsibility across the interprofessional team

Advocacy and action on infrastructural determinants should be distributed across the whole care team and its community partners, rather than added as an extra burden to physicians. Responsibility is allocated by proximity to the problem and capacity to intervene: physiotherapists are often first to identify unsafe stairs or lack of grab rails; pharmacists frequently detect transport barriers or non-collection; nurses and social workers can activate housing repairs or welfare routes; link workers connect patients to local authority and voluntary services; and recurrent or area-wide hazards trigger involvement from public health, housing, planning and engineering. To operationalize this, routine histories across professions include infrastructure red flags; a simple spot–route–act pathway sets who notices, who acts, and when to escalate; multidisciplinary team (MDT) huddles or inbox rules surface unresolved risks; and low-burden documentation (tick-boxes or smart phrases) limits cognitive load. Framed as team-based, pathway-driven advocacy, this approach keeps actions practical and time-bounded, aligns with existing scopes of practice, and mitigates burnout risk while ensuring that infrastructure is addressed when it is the proximal driver of harm.

## Future directions

With curricular and professional shifts defined, the next step is scaling and evaluation. Future educational strategies should prioritize curricular innovation, integrating practical advocacy training and interprofessional collaboration into clinical teaching. Rather than treating infrastructure as a peripheral concern, medical schools should commit to producing graduates who are systemic advocates capable of addressing structural determinants within their practice. Achieving meaningful curricular transformation will require the adoption of structured strategies, careful use of facilitators, and the removal of barriers to maximize the clinical impact of infrastructure-focused training (Table 2).

**Table 2:** Educational approaches to infrastructure training with barriers and facilitators.

**Educational approach**	**Description and purpose**	**Potential barriers**	**Facilitators and solutions**
Infrastructure-focused history taking	Routine clinical questioning about housing, utilities, transport to uncover infrastructural health determinants	Limited curricular time, perception as non-essential clinical skill	Clearly integrated curriculum modules, case studies demonstrating direct clinical relevance
Case-based learning scenarios	Clinical scenarios explicitly linking infrastructure deficits to patient presentations	Insufficient faculty awareness, lack of appropriate teaching resources	Structured teaching resources, faculty training, real-world case examples linking infrastructure to outcomes
Community-based clinical placements	Clinical rotations in environments visibly impacted by infrastructural deficits, e.g., disadvantaged urban areas, rural healthcare sites	Logistical challenges, limited placement availability, lack of institutional partnerships	Strong institutional support, established partnerships with community and governmental organizations
Interdisciplinary workshops and collaboration	Training involving collaborations with urban planners, engineers, public health professionals to broaden trainee perspectives	Organizational silos, limited cross-disciplinary communication	Formal interprofessional education programs, incentives for cross-disciplinary collaborations
Advocacy skills training	Explicit curriculum training students in advocacy, policy engagement, and stakeholder communication	Unclear professional role expectations, limited faculty experience, perceived irrelevance by some clinical educators	Dedicated advocacy curriculum units, clear professional guidelines supporting clinician advocacy, positive role modeling by senior clinicians

One potential future direction is the establishment of dedicated educational partnerships between medical schools, urban planners, housing authorities, and transport organizations. These partnerships could offer structured clinical placements designed explicitly to illustrate infrastructure-driven health outcomes, providing trainees first-hand experience in systemic advocacy. Similarly, integrating emerging digital technologies into medical training can help clinicians map and visualize infrastructure-related health inequalities, facilitating targeted interventions and advocacy.

Another approach is the creation of specialty clinical advocacy curricula with instruction on policy analysis, health effects assessment, and stakeholder communication. Practical advocacy training for students—how to interact effectively with policymakers, communicate complex clinical evidence, and enlist community collaborations—provides future clinicians with the necessary skills for effectuating structural change. Pilot curricula assessing the outcomes of such interventions on confidence for the trainee, patient health measures, and community effects might yield essential evidence for broader curricular implementation.

Ultimately, organizations should champion clinician-driven research into infrastructure as a clinical determinant through the systematized documentation of correlations between specified infrastructural interventions and measurable health benefits. Scaling up such research might establish robust evidence platforms to guide policy decisions and clinical practice guidelines where the direct clinical implications of infrastructure are accepted formally. Such research would additionally endorse the promotion of infrastructure as part of clinical practice with more organizational and professional support.

The future of medical education lies not in reinforcing outdated boundaries but in proactively redefining clinical roles to include infrastructure advocacy. Such proactive curricular and professional development prepares clinicians to better promote health, close gaps, and promote the built environments essential to the wellbeing of patients and communities.
